# Mitigating clinician and community concerns about children's flatfeet, intoed gait, or knock-knees

**DOI:** 10.1186/1757-1146-8-S2-O13

**Published:** 2015-09-22

**Authors:** Angela Evans

**Affiliations:** 1 Department of Podiatry, La Trobe University, Bundoora, Victoria, 3086, Australia; 2 Marion Podiatry, Oaklands Park, Adelaide, South Australia, 5046, Australia

**Keywords:** Children, flatfeet, intoed, knees

## Background

Foot and lower limb postures are a common parental concern, and present frequently to a range of clinicians. Referral of children with flatfeet, intoed gait, or knock-knees that is within normal developmental limits has been reported as approximating 40%. This represents a substantial and unnecessary cost to any health care system. The dilemma for caring clinicians is that each of these common presentations can be frankly pathological, so erring on the side of caution is understandable.

## Process

Review of current research for the evidence for treatment of each of flatfeet, intoed gait, or knock-knees/bow-legs, focussed on diagnosis. Intention was to reduce overuse of both referred (specialist) consultations and unnecessary intervention.

## Findings

It would appear that many clinicians are unsure as to the bounds of what is physiologically normal in terms of foot posture, gait angles, and knee position in childhood. The issue of intervention for paediatric flatfoot is repeatedly reported as being controversial. This need not be, given the direction from evidence and clinical guidelines. Gait angle of progression, and especially intoed gait, is a common concern for which there are known developmental patterns, and scant evidence for intervention. Knee position has a consistently documented progress with age, which generally guides the need for management.

## Conclusions

A framework for clinicians provides three quick questions that can clarify the need to attribute concern for each of these paediatric musculoskeletal conditions, and a diagnostic directive that will reduce the chance of overlooking something more serious – the *3qq*.

